# Supplementary material for: Long-term risk of autoimmune diseases after mRNA-based SARS-CoV2 vaccination in a Korean, nationwide, population-based cohort study
Source: Nat Commun. 2024 Jul 23;15:6181. doi: 10.1038/s41467-024-50656-8 (PMC11263712; doi:10.1038/s41467-024-50656-8)
Supplement: Supplementary file 1 — Supplementary Information [file 41467_2024_50656_MOESM1_ESM.pdf]

Supplementary Information

Long-Term Risk of Autoimmune Diseases After mRNA-based COVID-19 Vaccination: A Korean Nationwide Population-based Cohort Study

Seung-Won Jung[‡]; Jae Joon Jeon[‡]; You Hyun Kim; Sung Jay Choe[*]; Solam Lee[*]

Department of Dermatology, Yonsei University Wonju College of Medicine, Wonju, Republic of Korea

[‡] Contributed equally

[*] Correspondence authors

Corresponding Author: Solam Lee, M.D., Ph.D.

Email: solam@yonsei.ac.kr

Corresponding Author: Sung Jay Choe, M.D., Ph.D.

Email: wow8561@yonsei.ac.kr

List of Contents

- **Supplementary Figure 1.** Cumulative incidences of autoimmune connective tissue diseases and predefined positive and negative control outcomes
- **Supplementary Figure 2.** Stratified analyses of the risks of incident autoimmune connective tissue disorders in the mRNA-based COVID-19 vaccination cohort compared with the historical control cohort in the male subgroup
- **Supplementary Figure 3.** Stratified analyses of the risks of incident autoimmune connective tissue disorders in the mRNA-based COVID-19 vaccination cohort compared with the historical control cohort in the female subgroup
- **Supplementary Figure 4.** Stratified analyses of the risks of incident autoimmune connective tissue disorders in the mRNA-based COVID-19 vaccination cohort compared with the historical control cohort in the subgroup aged <40 years
- **Supplementary Figure 5.** Stratified analyses of the risks of incident autoimmune connective tissue disorders in the mRNA-based COVID-19 vaccination cohort compared with the historical control cohort in the subgroup aged ≥40 years
- **Supplementary Figure 6.** Stratified analyses of the risks of incident autoimmune connective tissue disorders in the mRNA-based COVID-19 vaccination cohort compared with the historical control cohort in the subgroup without COVID-19 diagnosis
- **Supplementary Figure 7.** Stratified analyses of the risks of incident autoimmune connective tissue disorders in the mRNA-based COVID-19 vaccination cohort compared with the historical control cohort in the subgroup with COVID-19 diagnosis
- **Supplementary Figure 8.** Stratified analyses of the risks of incident autoimmune connective tissue disorders in the mRNA-based COVID-19 vaccination cohort compared with the historical control cohort in the subgroup who received the BNT162b2 vaccine
- **Supplementary Figure 9.** Stratified analyses of the risks of incident autoimmune connective tissue disorders in the mRNA-based COVID-19 vaccination cohort compared with the historical control cohort in the subgroup who received the mRNA-1273 vaccine
- **Supplementary Figure 10.** Stratified analyses of the risks of incident autoimmune connective tissue disorders in the mRNA-based COVID-19 vaccination cohort compared with the historical control cohort in the subgroup who received only mRNA-based vaccines

- **Supplementary Figure 11.** Stratified analyses of the risks of incident autoimmune connective tissue disorders in the mRNA-based COVID-19 vaccination cohort compared with the historical control cohort in the subgroup who had history of cross-vaccination with any non-mRNA vaccines
- **Supplementary Table 1.** COVID-19 vaccination profiles of the vaccination cohort
- **Supplementary Table 2.** *International Statistical Classification of Diseases*, Tenth Revision (ICD-10) codes of the included diseases

Supplementary Figure 1. Cumulative incidences of autoimmune connective tissue diseases and predefined positive and negative control outcomes

The cumulative incidence plot shows the cumulative incidences of autoimmune connective tissue diseases and predefined positive and negative control outcomes, with the cumulative number of events for each cumulative event day in mRNA-based COVID-19 vaccination cohort and historical control cohort. The shaded area shows a 95% confidence interval for the cumulative incidences.

Abbreviation: ANCA, anti-neutrophil cytoplasmic antibody; COVID-19, coronavirus disease 2019.

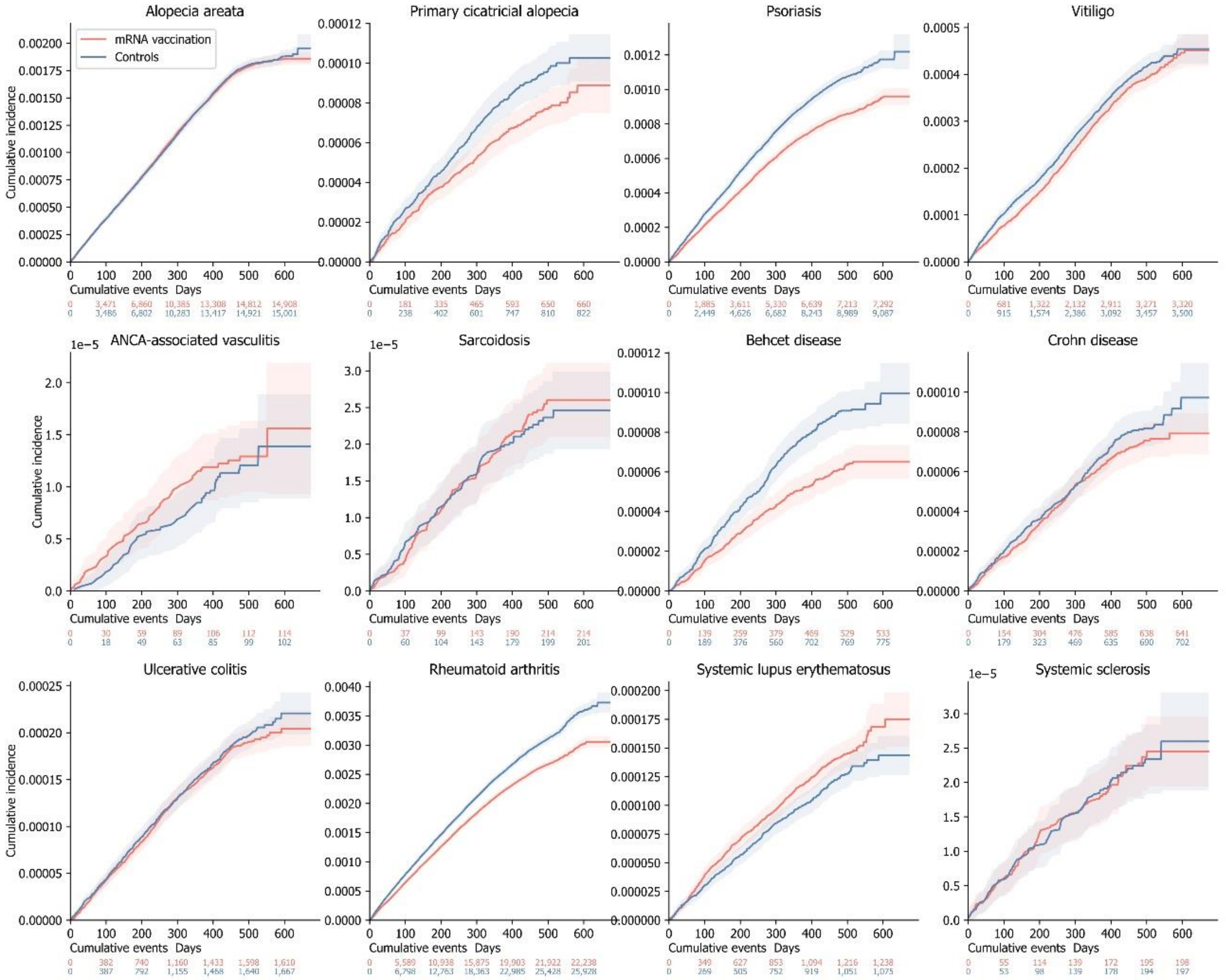

Supplementary Figure 1. Cumulative incidences of autoimmune connective tissue diseases and predefined positive and negative control outcomes (continued)

The cumulative incidence plot shows the cumulative incidences of autoimmune connective tissue diseases and predefined positive and negative control outcomes, with the cumulative number of events for each cumulative event day in mRNA-based COVID-19 vaccination cohort and historical control cohort. The shaded area shows a 95% confidence interval for the cumulative incidences.

Abbreviation: ANCA, anti-neutrophil cytoplasmic antibody; COVID-19, coronavirus disease 2019.

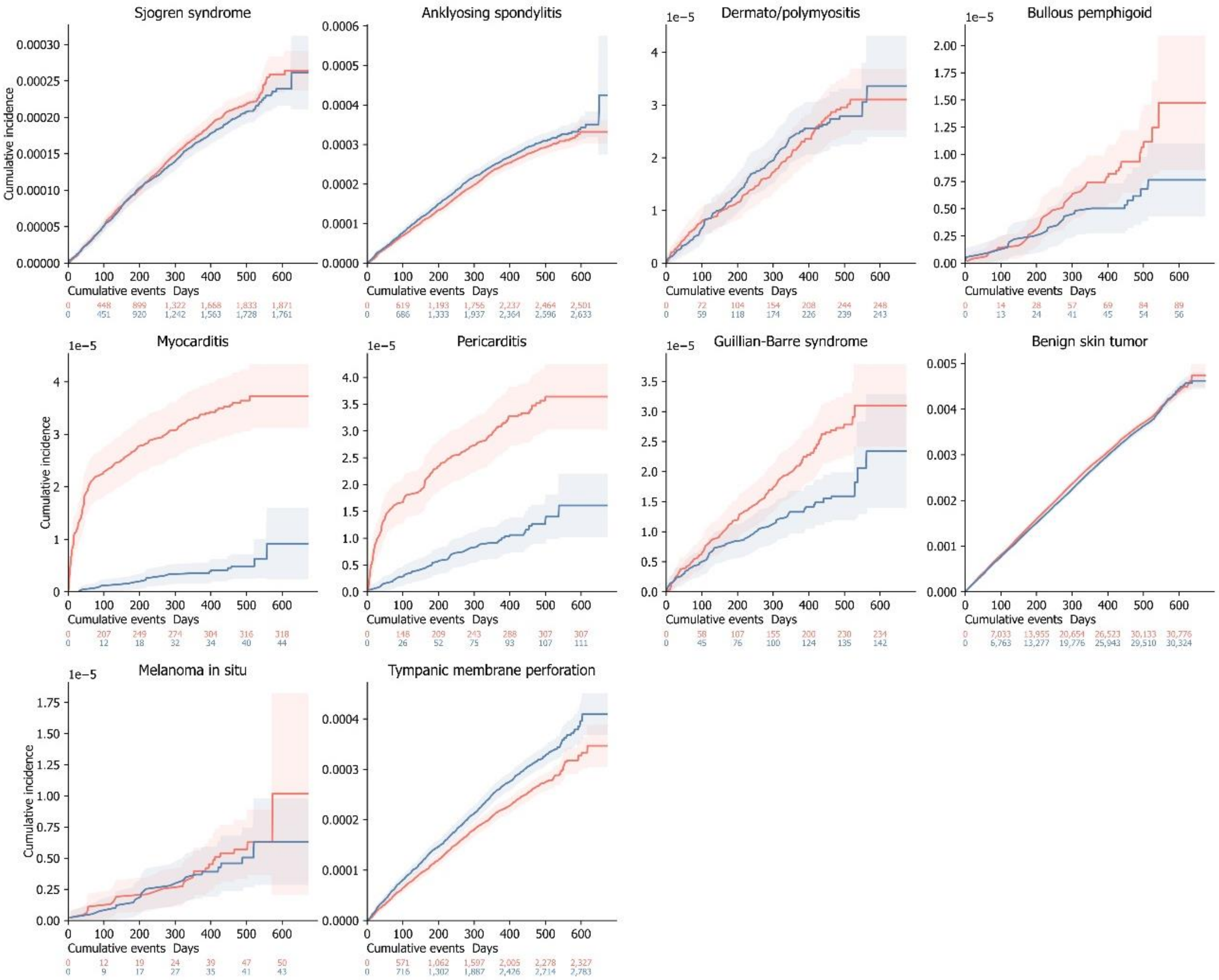

Supplementary Figure 2. Stratified analyses of the risks of incident autoimmune connective tissue disorders in the mRNA-based COVID-19 vaccination cohort compared with the historical control cohort in the male subgroup

The forest plot depicts adjusted hazard ratios (aHRs) with 99% confidence intervals (CIs) in the male individuals in the mRNA-based COVID-19 vaccination compared with historical controls. The point estimate (centre) represents the aHR, and the horizontal line (error bar) shows the range of the 99% CI. The incidence rate was calculated as the number of events divided by 10,000 person-years, with the population at risk also presented.

Abbreviation: aHR, adjusted hazard ratio; ANCA, antineutrophil cytoplasmic antibody; CI, confidence interval; COVID-19, coronavirus disease 2019; HR, hazard ratio.

	Incidence rate (no. of events/population at risk)			
	**mRNA Vaccination**	**Historical Controls**	HR (99% CI)	**aHR (99% CI)**
Autoimmune outcomes				
Alopecia areata	14.30 (4,308/2,354,725)	15.01 (4,538/2,362,417)	1.01 (0.96-1.05)	1.02 (0.97-1.06)
Cicatricial alopecia	0.65 (198/2,382,840)	0.82 (250/2,382,626)	0.78 (0.63-0.96)	0.78 (0.63-0.96)
Psoriasis	7.23 (2,181/2,356,189)	8.85 (2,677/2,362,284)	0.77 (0.72-0.82)	0.77 (0.72-0.82)
Vitiligo	2.38 (726/2,379,120)	2.46 (749/2,379,782)	0.92 (0.82-1.03)	0.92 (0.82-1.03)
ANCA-associated vasculitis	0.08 (25/2,384,607)	0.07 (21/2,383,847)	1.02 (0.53-1.97)	1.01 (0.52-1.94)
Sarcoidosis	0.19 (59/2,384,384)	0.16 (48/2,383,717)	1.16 (0.75-1.78)	1.15 (0.75-1.77)
Behcet disease	0.31 (95/2,383,007)	0.48 (148/2,382,549)	0.62 (0.46-0.83)	0.62 (0.46-0.82)
Crohn disease	0.66 (200/2,382,827)	0.74 (227/2,382,475)	0.88 (0.71-1.09)	0.88 (0.71-1.09)
Ulcerative colitis	1.69 (516/2,379,076)	1.69 (515/2,379,661)	1.01 (0.88-1.15)	1.00 (0.88-1.15)
Rheumatoid arthritis	15.04 (4,508/2,342,137)	15.55 (4,687/2,354,735)	0.88 (0.84-0.93)	0.88 (0.84-0.92)
Systemic lupus erythematosus	0.53 (163/2,383,691)	0.38 (116/2,383,222)	1.24 (0.95-1.61)	1.23 (0.94-1.60)
Systemic sclerosis	0.07 (22/2,384,600)	0.05 (15/2,383,853)	1.32 (0.64-2.72)	1.30 (0.63-2.69)
Sjogren syndrome	0.72 (221/2,383,264)	0.53 (162/2,382,936)	1.15 (0.92-1.44)	1.12 (0.90-1.41)
Anklyosing spondylitis	2.33 (710/2,377,574)	2.32 (708/2,378,305)	0.96 (0.85-1.08)	0.96 (0.85-1.08)
Dermato/polymyositis	0.19 (59/2,384,406)	0.20 (62/2,383,726)	0.84 (0.56-1.25)	0.83 (0.55-1.23)
Bullous pemphigoid	0.08 (23/2,384,677)	0.06 (17/2,383,920)	1.06 (0.53-2.14)	1.02 (0.51-2.05)
Positive control outcomes				
Myocarditis	0.24 (74/2,384,640)	0.04 (13/2,383,893)	5.28 (2.79-10.01)	5.26 (2.77-9.97)
Pericarditis	0.25 (76/2,384,555)	0.12 (37/2,383,833)	2.01 (1.30-3.12)	2.01 (1.30-3.12)
Guillian-Barre syndrome	0.25 (76/2,384,397)	0.14 (42/2,383,696)	1.69 (1.11-2.58)	1.68 (1.10-2.56)
Negative control outcomes				
Benign skin tumor	29.17 (8,732/2,341,541)	27.76 (8,371/2,357,744)	1.01 (0.98-1.04)	1.01 (0.97-1.04)
Melanoma in situ	0.04 (11/2,384,683)	0.03 (8/2,383,929)	1.33 (0.48-3.68)	1.32 (0.48-3.66)
Tympanic membrane perforation	1.91 (583/2,380,734)	2.18 (665/2,381,342)	0.81 (0.72-0.92)	0.81 (0.71-0.92)

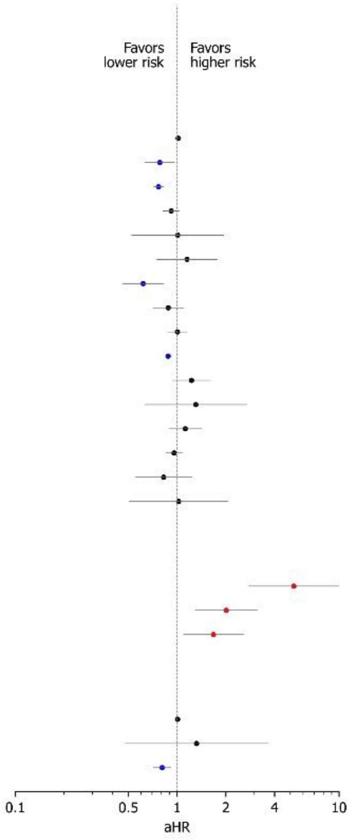

Supplementary Figure 3. Stratified analyses of the risks of incident autoimmune connective tissue disorders in the mRNA-based COVID-19 vaccination cohort compared with the historical control cohort in the female subgroup

The forest plot depicts adjusted hazard ratios (aHRs) with 99% confidence intervals (CIs) in the female individuals in the mRNA-based COVID-19 vaccination compared with historical controls. The point estimate (centre) represents the aHR, and the horizontal line (error bar) shows the range of the 99% CI. The incidence rate was calculated as the number of events divided by 10,000 person-years, with the population at risk also presented.

Abbreviation: aHR, adjusted hazard ratio; ANCA, antineutrophil cytoplasmic antibody; CI, confidence interval; COVID-19, coronavirus disease 2019; HR, hazard ratio.

	Incidence rate (no. of events/population at risk) mRNA Vaccination	Historical Controls	HR (99% CI)	aHR (99% CI)
Autoimmune outcomes				
Alopecia areata	11.31 (3,000/2,040,106)	11.95 (3,179/2,046,554)	0.98 (0.93-1.04)	0.98 (0.92-1.03)
Cicatricial alopecia	0.50 (133/2,059,451)	0.60 (161/2,060,188)	0.81 (0.63-1.05)	0.81 (0.63-1.05)
Psoriasis	5.92 (1,572/2,043,566)	6.66 (1,773/2,048,113)	0.86 (0.80-0.93)	0.86 (0.79-0.93)
Vitiligo	3.68 (983/2,054,016)	3.57 (954/2,056,126)	0.97 (0.87-1.07)	0.97 (0.88-1.07)
ANCA-associated vasculitis	0.13 (35/2,060,422)	0.11 (29/2,060,859)	1.14 (0.66-1.99)	1.15 (0.66-2.00)
Sarcoidosis	0.19 (52/2,060,197)	0.18 (49/2,060,630)	0.97 (0.63-1.51)	0.97 (0.63-1.51)
Behcet disease	0.67 (180/2,058,014)	0.87 (232/2,058,730)	0.74 (0.59-0.91)	0.74 (0.59-0.92)
Crohn disease	0.48 (128/2,059,702)	0.46 (123/2,060,354)	0.98 (0.74-1.30)	0.98 (0.74-1.30)
Ulcerative colitis	1.09 (291/2,057,464)	1.21 (325/2,058,473)	0.90 (0.76-1.08)	0.90 (0.76-1.08)
Rheumatoid arthritis	27.73 (7,128/1,979,315)	29.68 (7,712/2,001,237)	0.85 (0.82-0.88)	0.85 (0.82-0.88)
Systemic lupus erythematosus	1.81 (483/2,056,117)	1.51 (404/2,057,602)	1.13 (0.97-1.31)	1.13 (0.98-1.31)
Systemic sclerosis	0.30 (81/2,059,898)	0.29 (77/2,060,414)	0.94 (0.66-1.33)	0.94 (0.66-1.34)
Sjogren syndrome	2.91 (777/2,054,611)	2.50 (669/2,056,642)	1.04 (0.93-1.17)	1.05 (0.93-1.18)
Anklyosing spondylitis	2.18 (584/2,056,394)	2.14 (572/2,058,077)	0.94 (0.82-1.07)	0.94 (0.83-1.07)
Dermato/polymyositis	0.26 (70/2,060,180)	0.21 (55/2,060,670)	1.24 (0.84-1.84)	1.25 (0.84-1.85)
Bullous pemphigoid	0.10 (26/2,060,524)	0.03 (8/2,060,948)	2.76 (1.15-6.63)	2.67 (1.11-6.42)
Positive control outcomes				
Myocarditis	0.33 (89/2,060,517)	0.03 (8/2,060,937)	10.54 (4.68-23.73)	10.53 (4.68-23.72)
Pericarditis	0.29 (79/2,060,495)	0.06 (17/2,060,901)	4.29 (2.41-7.63)	4.30 (2.42-7.64)
Guillian-Barre syndrome	0.18 (47/2,060,382)	0.11 (29/2,060,826)	1.53 (0.91-2.59)	1.54 (0.91-2.60)
Negative control outcomes				
Benign skin tumor	26.60 (7,001/2,027,286)	24.66 (6,536/2,040,957)	1.04 (1.00-1.08)	1.04 (1.00-1.08)
Melanoma in situ	0.06 (16/2,060,519)	0.04 (12/2,060,937)	1.14 (0.50-2.60)	1.15 (0.50-2.61)
Tympanic membrane perforation	2.37 (633/2,056,393)	2.56 (685/2,058,016)	0.86 (0.76-0.97)	0.86 (0.76-0.97)

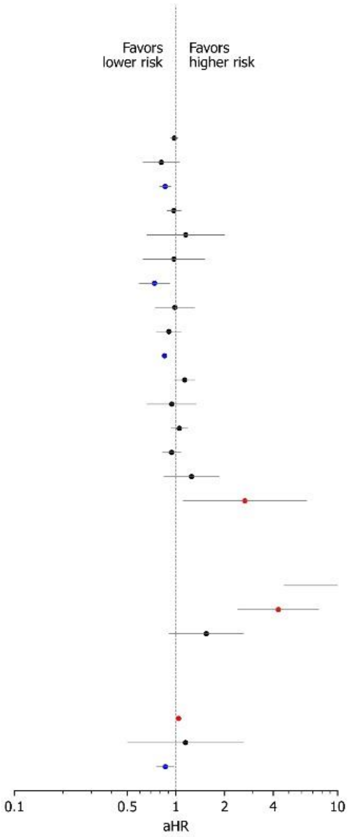

Supplementary Figure 4. Stratified analyses of the risks of incident autoimmune connective tissue disorders in the mRNA-based COVID-19 vaccination cohort compared with the historical control cohort in the subgroup aged <40 years

The forest plot depicts adjusted hazard ratios (aHRs) with 99% confidence intervals (CIs) in the individuals aged <40 years in the mRNA-based COVID-19 vaccination compared with historical controls. The point estimate (centre) represents the aHR, and the horizontal line (error bar) shows the range of the 99% CI. The incidence rate was calculated as the number of events divided by 10,000 person-years, with the population at risk also presented.

Abbreviation: aHR, adjusted hazard ratio; ANCA, antineutrophil cytoplasmic antibody; CI, confidence interval; COVID-19, coronavirus disease 2019; HR, hazard ratio.

	Incidence rate (no. of events/population at risk)			
	**mRNA Vaccination**	**Historical Controls**	HR (99% CI)	**aHR (99% CI)**
Autoimmune outcomes				
Alopecia areata	19.39 (1,977/791,782)	19.66 (2,435/962,576)	0.98 (0.91-1.04)	1.00 (0.93-1.07)
Cicatricial alopecia	0.75 (78/803,473)	0.93 (117/972,414)	0.81 (0.59-1.11)	0.84 (0.61-1.16)
Psoriasis	5.78 (594/797,930)	6.88 (857/967,405)	0.80 (0.71-0.90)	0.80 (0.71-0.90)
Vitiligo	2.14 (222/802,733)	1.94 (243/971,640)	1.09 (0.88-1.33)	1.08 (0.88-1.32)
ANCA-associated vasculitis	0.04 (4/804,194)	0.05 (6/972,961)	0.82 (0.20-3.38)	0.79 (0.19-3.28)
Sarcoidosis	0.13 (13/804,133)	0.14 (18/972,906)	0.85 (0.38-1.88)	0.88 (0.39-1.95)
Behcet disease	0.43 (45/803,829)	0.56 (70/972,580)	0.74 (0.49-1.12)	0.69 (0.46-1.05)
Crohn disease	0.79 (82/803,315)	0.83 (104/972,149)	0.94 (0.68-1.29)	0.96 (0.70-1.34)
Ulcerative colitis	1.89 (196/802,737)	1.77 (222/971,722)	1.06 (0.85-1.31)	1.08 (0.87-1.34)
Rheumatoid arthritis	9.00 (925/797,828)	8.72 (1,087/967,755)	0.99 (0.90-1.09)	0.96 (0.87-1.06)
Systemic lupus erythematosus	0.95 (98/803,510)	0.74 (93/972,326)	1.18 (0.86-1.63)	1.09 (0.79-1.50)
Systemic sclerosis	0.08 (8/804,187)	0.05 (6/972,944)	1.53 (0.46-5.06)	1.36 (0.41-4.51)
Sjogren syndrome	0.55 (57/803,874)	0.49 (62/972,697)	1.05 (0.70-1.57)	0.97 (0.64-1.45)
Anklyosing spondylitis	2.28 (236/802,539)	1.93 (241/971,341)	1.14 (0.93-1.39)	1.15 (0.94-1.41)
Dermato/polymyositis	0.13 (13/804,162)	0.08 (10/972,937)	1.54 (0.59-4.00)	1.43 (0.55-3.71)
Bullous pemphigoid	0.00 (0/804,216)	0.00 (0/972,977)		
Positive control outcomes				
Myocarditis	0.40 (41/804,189)	0.03 (4/972,964)	12.59 (3.73-42.49)	12.53 (3.71-42.35)
Pericarditis	0.38 (39/804,181)	0.06 (8/972,953)	5.64 (2.35-13.50)	5.39 (2.25-12.94)
Guillian-Barre syndrome	0.10 (10/804,171)	0.09 (11/972,929)	1.09 (0.42-2.86)	1.06 (0.40-2.78)
Negative control outcomes				
Benign skin tumor	23.79 (2,431/793,542)	21.95 (2,727/965,538)	1.07 (1.01-1.14)	1.07 (1.01-1.14)
Melanoma in situ	0.01 (1/804,209)	0.01 (1/972,973)	1.21 (0.05-27.11)	1.35 (0.06-30.60)
Tympanic membrane perforation	1.03 (107/803,498)	1.20 (150/972,409)	0.85 (0.65-1.13)	0.85 (0.64-1.11)

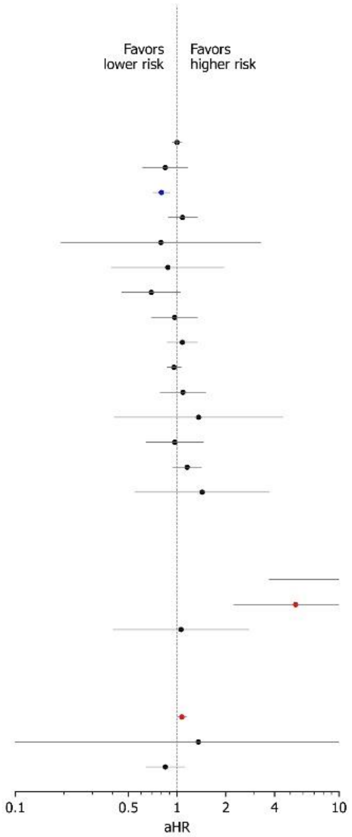

Supplementary Figure 5. Stratified analyses of the risks of incident autoimmune connective tissue disorders in the mRNA-based COVID-19 vaccination cohort compared with the historical control cohort in the subgroup aged ≥40 years

The forest plot depicts adjusted hazard ratios (aHRs) with 99% confidence intervals (CIs) in the individuals aged ≥40 years in the mRNA-based COVID-19 vaccination compared with historical controls. The point estimate (centre) represents the aHR, and the horizontal line (error bar) shows the range of the 99% CI. The incidence rate was calculated as the number of events divided by 10,000 person-years, with the population at risk also presented.

Abbreviation: aHR, adjusted hazard ratio; ANCA, antineutrophil cytoplasmic antibody; CI, confidence interval; COVID-19, coronavirus disease 2019; HR, hazard ratio.

	Incidence rate (no. of events/population at risk)			
	**mRNA Vaccination**	**Historical Controls**	HR (99% CI)	**aHR (99% CI)**
Autoimmune outcomes				
Alopecia areata	11.48 (5,331/3,603,049)	11.88 (5,282/3,446,395)	1.00 (0.96-1.04)	1.00 (0.95-1.04)
Cicatricial alopecia	0.54 (253/3,638,818)	0.66 (294/3,470,400)	0.79 (0.65-0.95)	0.79 (0.65-0.95)
Psoriasis	6.80 (3,159/3,601,825)	8.09 (3,593/3,442,992)	0.80 (0.76-0.85)	0.80 (0.76-0.85)
Vitiligo	3.17 (1,487/3,630,403)	3.27 (1,460/3,464,268)	0.93 (0.86-1.01)	0.93 (0.86-1.01)
ANCA-associated vasculitis	0.12 (56/3,640,835)	0.10 (44/3,471,745)	1.13 (0.72-1.75)	1.12 (0.72-1.75)
Sarcoidosis	0.21 (98/3,640,448)	0.18 (79/3,471,441)	1.11 (0.79-1.54)	1.11 (0.79-1.54)
Behcet disease	0.49 (230/3,637,192)	0.69 (310/3,468,699)	0.68 (0.56-0.83)	0.68 (0.56-0.83)
Crohn disease	0.52 (246/3,639,214)	0.55 (246/3,470,680)	0.91 (0.75-1.11)	0.91 (0.74-1.11)
Ulcerative colitis	1.30 (611/3,633,803)	1.38 (618/3,466,412)	0.94 (0.83-1.06)	0.93 (0.82-1.06)
Rheumatoid arthritis	23.60 (10,711/3,523,624)	25.91 (11,312/3,388,217)	0.85 (0.83-0.88)	0.85 (0.83-0.88)
Systemic lupus erythematosus	1.17 (548/3,636,298)	0.95 (427/3,468,498)	1.15 (1.00-1.32)	1.16 (1.00-1.33)
Systemic sclerosis	0.20 (95/3,640,311)	0.19 (86/3,471,323)	0.97 (0.70-1.35)	0.98 (0.71-1.36)
Sjogren syndrome	2.01 (941/3,634,001)	1.72 (769/3,466,881)	1.07 (0.96-1.19)	1.07 (0.96-1.19)
Anklyosing spondylitis	2.26 (1,058/3,631,429)	2.32 (1,039/3,465,041)	0.91 (0.83-1.00)	0.91 (0.83-1.00)
Dermato/polymyositis	0.25 (116/3,640,424)	0.24 (107/3,471,459)	0.98 (0.73-1.32)	0.99 (0.73-1.32)
Bullous pemphigoid	0.10 (49/3,640,985)	0.06 (25/3,471,891)	1.59 (0.93-2.71)	1.53 (0.90-2.61)
Positive control outcomes				
Myocarditis	0.26 (122/3,640,968)	0.04 (17/3,471,866)	6.16 (3.55-10.68)	6.18 (3.56-10.71)
Pericarditis	0.25 (116/3,640,869)	0.10 (46/3,471,781)	2.30 (1.57-3.36)	2.30 (1.57-3.36)
Guillian-Barre syndrome	0.24 (113/3,640,608)	0.13 (60/3,471,593)	1.71 (1.20-2.43)	1.70 (1.20-2.42)
Negative control outcomes				
Benign skin tumor	28.89 (13,302/3,575,285)	27.54 (12,180/3,433,163)	1.01 (0.98-1.04)	1.01 (0.98-1.04)
Melanoma in situ	0.06 (26/3,640,993)	0.04 (19/3,471,893)	1.21 (0.63-2.33)	1.21 (0.63-2.34)
Tympanic membrane perforation	2.37 (1,109/3,633,629)	2.68 (1,200/3,466,949)	0.84 (0.76-0.92)	0.83 (0.76-0.92)

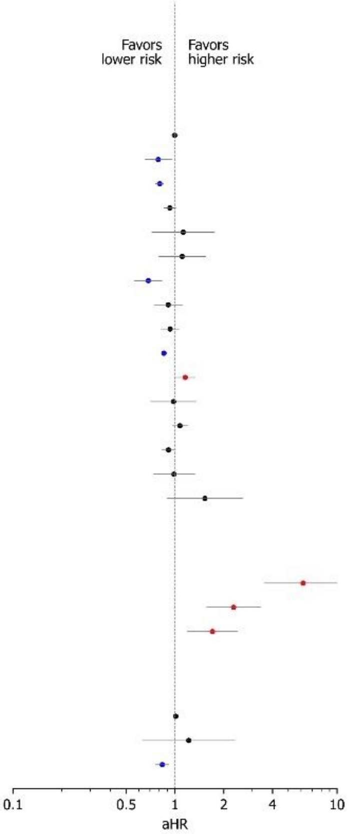

Supplementary Figure 6. Stratified analyses of the risks of incident autoimmune connective tissue disorders in the mRNA-based COVID-19 vaccination cohort compared with the historical control cohort in the subgroup without COVID-19 diagnosis

The forest plot depicts adjusted hazard ratios (aHRs) with 99% confidence intervals (CIs) in the individuals without COVID-19 diagnosis in the mRNA-based COVID-19 vaccination compared with historical controls. The point estimate (centre) represents the aHR, and the horizontal line (error bar) shows the range of the 99% CI. The incidence rate was calculated as the number of events divided by 10,000 person-years, with the population at risk also presented.

Abbreviation: aHR, adjusted hazard ratio; ANCA, antineutrophil cytoplasmic antibody; CI, confidence interval; COVID-19, coronavirus disease 2019; HR, hazard ratio.

	Incidence rate (no. of events/population at risk)			
	**mRNA Vaccination**	**Historical Controls**	HR (99% CI)	**aHR (99% CI)**
Autoimmune outcomes				
Alopecia areata	11.68 (3,554/2,360,680)	13.58 (7,717/4,408,971)	0.90 (0.86-0.94)	0.97 (0.92-1.01)
Cicatricial alopecia	0.55 (170/2,384,225)	0.72 (411/4,442,814)	0.76 (0.62-0.93)	0.78 (0.63-0.95)
Psoriasis	7.01 (2,134/2,360,803)	7.82 (4,450/4,410,397)	0.85 (0.80-0.90)	0.83 (0.78-0.88)
Vitiligo	2.90 (891/2,379,529)	2.98 (1,703/4,435,908)	0.92 (0.84-1.01)	0.90 (0.82-0.98)
ANCA-associated vasculitis	0.13 (40/2,385,674)	0.09 (50/4,444,706)	1.35 (0.84-2.16)	1.24 (0.77-1.99)
Sarcoidosis	0.19 (58/2,385,438)	0.17 (97/4,444,347)	1.03 (0.71-1.49)	1.01 (0.70-1.46)
Behcet disease	0.38 (118/2,383,717)	0.66 (380/4,441,279)	0.55 (0.44-0.70)	0.56 (0.45-0.71)
Crohn disease	0.50 (154/2,384,501)	0.61 (350/4,442,829)	0.80 (0.64-0.99)	0.79 (0.64-0.98)
Ulcerative colitis	1.28 (393/2,381,421)	1.47 (840/4,438,134)	0.87 (0.76-1.00)	0.88 (0.77-1.00)
Rheumatoid arthritis	20.66 (6,167/2,318,171)	22.09 (12,399/4,355,972)	0.85 (0.82-0.88)	0.81 (0.78-0.84)
Systemic lupus erythematosus	1.03 (318/2,382,982)	0.91 (520/4,440,824)	1.06 (0.91-1.24)	1.09 (0.93-1.28)
Systemic sclerosis	0.19 (59/2,385,351)	0.16 (92/4,444,267)	1.06 (0.73-1.54)	1.06 (0.73-1.53)
Sjogren syndrome	1.75 (537/2,381,671)	1.45 (831/4,439,578)	1.08 (0.95-1.22)	1.04 (0.92-1.17)
Anklyosing spondylitis	2.15 (660/2,379,908)	2.24 (1,280/4,436,382)	0.90 (0.81-1.01)	0.87 (0.78-0.97)
Dermato/polymyositis	0.21 (66/2,385,412)	0.20 (117/4,444,396)	0.98 (0.70-1.38)	0.92 (0.65-1.29)
Bullous pemphigoid	0.09 (27/2,385,764)	0.04 (25/4,444,868)	1.65 (0.89-3.03)	1.32 (0.72-2.44)
Positive control outcomes				
Myocarditis	0.19 (58/2,385,736)	0.04 (21/4,444,830)	4.70 (2.70-8.18)	4.83 (2.77-8.41)
Pericarditis	0.24 (74/2,385,682)	0.09 (54/4,444,734)	2.48 (1.68-3.67)	2.46 (1.66-3.65)
Guillian-Barre syndrome	0.19 (57/2,385,516)	0.12 (71/4,444,522)	1.45 (0.98-2.14)	1.35 (0.91-2.00)
Negative control outcomes				
Benign skin tumor	27.99 (8,445/2,344,522)	26.31 (14,907/4,398,701)	1.02 (0.99-1.05)	1.00 (0.97-1.03)
Melanoma in situ	0.05 (14/2,385,764)	0.03 (20/4,444,866)	1.18 (0.55-2.53)	1.12 (0.52-2.40)
Tympanic membrane perforation	2.22 (682/2,381,459)	2.36 (1,350/4,439,358)	0.87 (0.79-0.97)	0.82 (0.74-0.92)

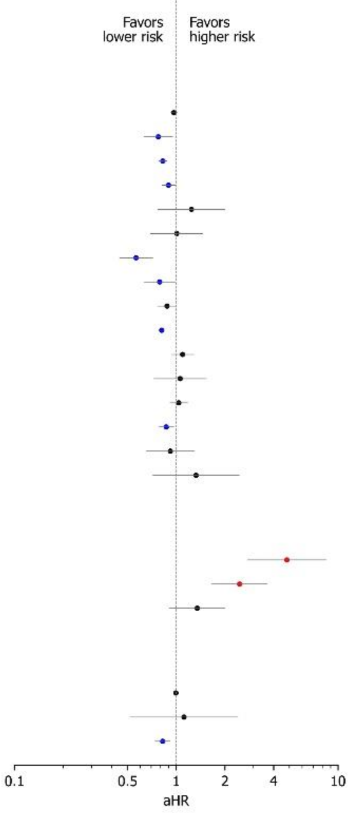

Supplementary Figure 7. Stratified analyses of the risks of incident autoimmune connective tissue disorders in the mRNA-based COVID-19 vaccination cohort compared with the historical control cohort in the subgroup with COVID-19 diagnosis

The forest plot depicts adjusted hazard ratios (aHRs) with 99% confidence intervals (CIs) in the individuals with COVID-19 diagnosis in the mRNA-based COVID-19 vaccination compared with historical controls. The point estimate (centre) represents the aHR, and the horizontal line (error bar) shows the range of the 99% CI. The incidence rate was calculated as the number of events divided by 10,000 person-years, with the population at risk also presented.

Abbreviation: aHR, adjusted hazard ratio; ANCA, antineutrophil cytoplasmic antibody; CI, confidence interval; COVID-19, coronavirus disease 2019; HR, hazard ratio.

	Incidence rate (no. of events/population at risk)			
	**mRNA Vaccination**	**Historical Controls**	HR (99% CI)	**aHR (99% CI)**
Autoimmune outcomes				
Alopecia areata	14.31 (3,754/2,034,151)	13.58 (7,717/4,408,971)	1.10 (1.05-1.15)	1.03 (0.99-1.08)
Cicatricial alopecia	0.61 (161/2,058,066)	0.72 (411/4,442,814)	0.83 (0.68-1.02)	0.82 (0.67-1.00)
Psoriasis	6.15 (1,619/2,038,952)	7.82 (4,450/4,410,397)	0.75 (0.70-0.80)	0.78 (0.73-0.83)
Vitiligo	3.09 (818/2,053,607)	2.98 (1,703/4,435,908)	0.98 (0.89-1.08)	1.01 (0.92-1.11)
ANCA-associated vasculitis	0.08 (20/2,059,355)	0.09 (50/4,444,706)	0.80 (0.45-1.43)	0.88 (0.49-1.59)
Sarcoidosis	0.20 (53/2,059,143)	0.17 (97/4,444,347)	1.11 (0.76-1.61)	1.12 (0.77-1.64)
Behcet disease	0.59 (157/2,057,304)	0.66 (380/4,441,279)	0.85 (0.69-1.05)	0.84 (0.68-1.03)
Crohn disease	0.65 (174/2,058,028)	0.61 (350/4,442,829)	1.06 (0.86-1.30)	1.06 (0.86-1.30)
Ulcerative colitis	1.56 (414/2,055,119)	1.47 (840/4,438,134)	1.07 (0.94-1.22)	1.06 (0.93-1.21)
Rheumatoid arthritis	21.17 (5,469/2,003,281)	22.09 (12,399/4,355,972)	0.87 (0.84-0.90)	0.93 (0.89-0.96)
Systemic lupus erythematosus	1.24 (328/2,056,826)	0.91 (520/4,440,824)	1.26 (1.08-1.47)	1.23 (1.05-1.44)
Systemic sclerosis	0.17 (44/2,059,147)	0.16 (92/4,444,267)	0.94 (0.63-1.40)	0.94 (0.63-1.41)
Sjogren syndrome	1.74 (461/2,056,204)	1.45 (831/4,439,578)	1.05 (0.92-1.20)	1.11 (0.98-1.26)
Anklyosing spondylitis	2.39 (634/2,054,060)	2.24 (1,280/4,436,382)	1.00 (0.90-1.12)	1.04 (0.94-1.16)
Dermato/polymyositis	0.24 (63/2,059,174)	0.20 (117/4,444,396)	1.08 (0.76-1.52)	1.16 (0.82-1.64)
Bullous pemphigoid	0.08 (22/2,059,437)	0.04 (25/4,444,868)	1.52 (0.79-2.89)	1.85 (0.97-3.54)
Positive control outcomes				
Myocarditis	0.39 (105/2,059,421)	0.04 (21/4,444,830)	10.03 (5.99-16.80)	9.97 (5.95-16.72)
Pericarditis	0.30 (81/2,059,368)	0.09 (54/4,444,734)	3.06 (2.08-4.49)	3.26 (2.21-4.79)
Guillian-Barre syndrome	0.25 (66/2,059,263)	0.12 (71/4,444,522)	1.84 (1.25-2.69)	2.01 (1.37-2.95)
Negative control outcomes				
Benign skin tumor	27.94 (7,288/2,024,305)	26.31 (14,907/4,398,701)	1.02 (0.99-1.05)	1.05 (1.02-1.09)
Melanoma in situ	0.05 (13/2,059,438)	0.03 (20/4,444,866)	1.24 (0.57-2.71)	1.35 (0.62-2.97)
Tympanic membrane perforation	2.01 (534/2,055,668)	2.36 (1,350/4,439,358)	0.79 (0.71-0.89)	0.85 (0.76-0.95)

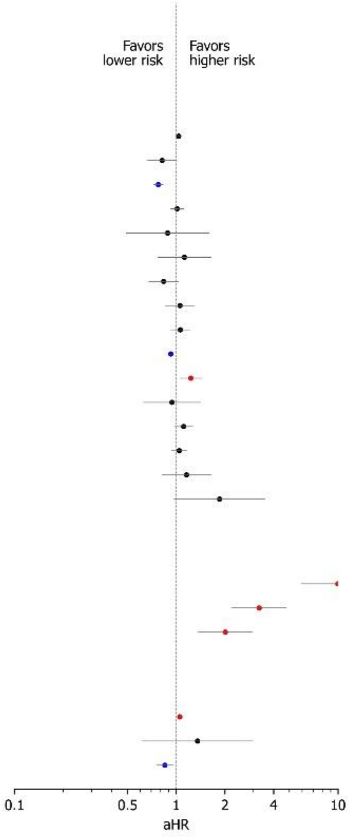

Supplementary Figure 8. Stratified analyses of the risks of incident autoimmune connective tissue disorders in the mRNA-based COVID-19 vaccination cohort compared with the historical control cohort in the subgroup who received the BNT162b2 vaccine

The forest plot depicts adjusted hazard ratios (aHRs) with 99% confidence intervals (CIs) in the individuals who received the BNT162b2 vaccine in the mRNA-based COVID-19 vaccination compared with historical controls. The point estimate (centre) represents the aHR, and the horizontal line (error bar) shows the range of the 99% CI. The incidence rate was calculated as the number of events divided by 10,000 person-years, with the population at risk also presented.

Abbreviation: aHR, adjusted hazard ratio; ANCA, antineutrophil cytoplasmic antibody; CI, confidence interval; COVID-19, coronavirus disease 2019; HR, hazard ratio.

	Incidence rate (no. of events/population at risk)			
	**mRNA Vaccination**	**Historical Controls**	HR (99% CI)	**aHR (99% CI)**
Autoimmune outcomes				
Alopecia areata	13.09 (5,095/2,887,570)	13.58 (7,717/4,408,971)	1.03 (0.99-1.07)	1.01 (0.97-1.05)
Cicatricial alopecia	0.58 (230/2,919,085)	0.72 (411/4,442,814)	0.81 (0.68-0.97)	0.81 (0.68-0.98)
Psoriasis	6.69 (2,608/2,891,892)	7.82 (4,450/4,410,397)	0.83 (0.78-0.87)	0.84 (0.80-0.89)
Vitiligo	2.95 (1,161/2,913,206)	2.98 (1,703/4,435,908)	0.95 (0.87-1.03)	0.95 (0.87-1.03)
ANCA-associated vasculitis	0.10 (39/2,920,884)	0.09 (50/4,444,706)	1.04 (0.65-1.68)	1.05 (0.65-1.69)
Sarcoidosis	0.20 (77/2,920,620)	0.17 (97/4,444,347)	1.09 (0.78-1.53)	1.10 (0.79-1.55)
Behcet disease	0.52 (205/2,918,183)	0.66 (380/4,441,279)	0.76 (0.63-0.92)	0.75 (0.62-0.91)
Crohn disease	0.59 (234/2,919,216)	0.61 (350/4,442,829)	0.97 (0.80-1.16)	0.98 (0.81-1.18)
Ulcerative colitis	1.46 (574/2,915,352)	1.47 (840/4,438,134)	1.02 (0.90-1.15)	1.03 (0.91-1.15)
Rheumatoid arthritis	21.06 (8,063/2,842,419)	22.09 (12,399/4,355,972)	0.88 (0.85-0.90)	0.88 (0.85-0.91)
Systemic lupus erythematosus	1.16 (456/2,917,282)	0.91 (520/4,440,824)	1.20 (1.04-1.38)	1.18 (1.02-1.36)
Systemic sclerosis	0.19 (76/2,920,545)	0.16 (92/4,444,267)	1.08 (0.76-1.51)	1.06 (0.75-1.49)
Sjogren syndrome	1.74 (684/2,916,018)	1.45 (831/4,439,578)	1.07 (0.96-1.20)	1.06 (0.95-1.19)
Anklyosing spondylitis	2.35 (925/2,913,579)	2.24 (1,280/4,436,382)	1.00 (0.91-1.10)	1.02 (0.92-1.12)
Dermato/polymyositis	0.21 (84/2,920,613)	0.20 (117/4,444,396)	0.97 (0.70-1.33)	0.99 (0.72-1.35)
Bullous pemphigoid	0.10 (40/2,921,014)	0.04 (25/4,444,868)	1.81 (1.04-3.16)	1.59 (0.91-2.80)
Positive control outcomes				
Myocarditis	0.28 (112/2,920,994)	0.04 (21/4,444,830)	7.32 (4.38-12.23)	7.29 (4.36-12.19)
Pericarditis	0.30 (118/2,920,921)	0.09 (54/4,444,734)	3.09 (2.16-4.42)	3.13 (2.19-4.49)
Guillian-Barre syndrome	0.25 (97/2,920,734)	0.12 (71/4,444,522)	1.88 (1.33-2.65)	1.91 (1.35-2.70)
Negative control outcomes				
Benign skin tumor	27.98 (10,821/2,872,156)	26.31 (14,907/4,398,701)	1.02 (1.00-1.05)	1.04 (1.01-1.06)
Melanoma in situ	0.05 (20/2,921,016)	0.03 (20/4,444,866)	1.26 (0.63-2.52)	1.25 (0.62-2.50)
Tympanic membrane perforation	2.15 (844/2,915,864)	2.36 (1,350/4,439,358)	0.85 (0.77-0.93)	0.86 (0.78-0.94)

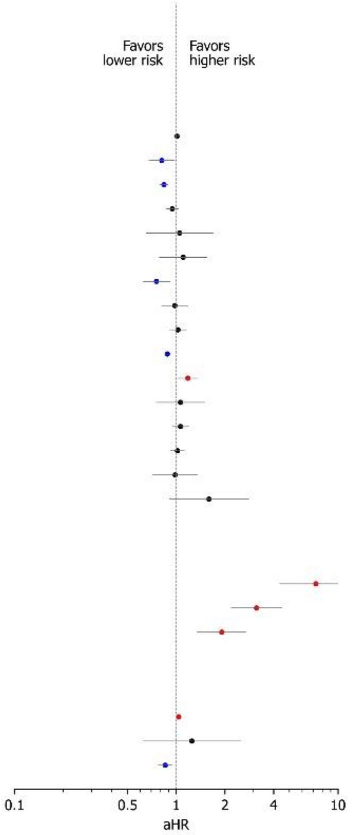

Supplementary Figure 9. Stratified analyses of the risks of incident autoimmune connective tissue disorders in the mRNA-based COVID-19 vaccination cohort compared with the historical control cohort in the subgroup who received the mRNA-1273 vaccine

The forest plot depicts adjusted hazard ratios (aHRs) with 99% confidence intervals (CIs) in the individuals who received the mRNA-1273 in the mRNA-based COVID-19 vaccination compared with historical controls. The point estimate (centre) represents the aHR, and the horizontal line (error bar) shows the range of the 99% CI. The incidence rate was calculated as the number of events divided by 10,000 person-years, with the population at risk also presented.

Abbreviation: aHR, adjusted hazard ratio; ANCA, antineutrophil cytoplasmic antibody; CI, confidence interval; COVID-19, coronavirus disease 2019; HR, hazard ratio.

	Incidence rate (no. of events/population at risk)			
	**mRNA Vaccination**	**Historical Controls**	HR (99% CI)	**aHR (99% CI)**
Autoimmune outcomes				
Alopecia areata	12.27 (2,067/1,440,198)	13.58 (7,717/4,408,971)	0.91 (0.87-0.96)	0.96 (0.91-1.02)
Cicatricial alopecia	0.56 (96/1,455,268)	0.72 (411/4,442,814)	0.75 (0.59-0.97)	0.75 (0.58-0.96)
Psoriasis	6.44 (1,086/1,440,529)	7.82 (4,450/4,410,397)	0.75 (0.70-0.81)	0.73 (0.67-0.78)
Vitiligo	3.08 (524/1,452,107)	2.98 (1,703/4,435,908)	0.95 (0.85-1.06)	0.95 (0.85-1.06)
ANCA-associated vasculitis	0.12 (21/1,456,158)	0.09 (50/4,444,706)	1.26 (0.70-2.27)	1.25 (0.69-2.25)
Sarcoidosis	0.19 (33/1,455,986)	0.17 (97/4,444,347)	1.04 (0.66-1.62)	1.00 (0.64-1.57)
Behcet disease	0.38 (65/1,454,925)	0.66 (380/4,441,279)	0.52 (0.38-0.70)	0.53 (0.39-0.72)
Crohn disease	0.53 (90/1,455,370)	0.61 (350/4,442,829)	0.82 (0.63-1.07)	0.81 (0.62-1.05)
Ulcerative colitis	1.27 (217/1,453,336)	1.47 (840/4,438,134)	0.85 (0.71-1.00)	0.83 (0.70-0.99)
Rheumatoid arthritis	20.60 (3,407/1,412,559)	22.09 (12,399/4,355,972)	0.83 (0.79-0.86)	0.81 (0.78-0.85)
Systemic lupus erythematosus	1.04 (178/1,454,622)	0.91 (520/4,440,824)	1.03 (0.85-1.25)	1.08 (0.89-1.32)
Systemic sclerosis	0.15 (26/1,455,978)	0.16 (92/4,444,267)	0.86 (0.53-1.41)	0.89 (0.54-1.45)
Sjogren syndrome	1.72 (293/1,453,963)	1.45 (831/4,439,578)	1.03 (0.88-1.20)	1.05 (0.90-1.22)
Anklyosing spondylitis	2.06 (350/1,452,606)	2.24 (1,280/4,436,382)	0.84 (0.74-0.96)	0.81 (0.71-0.93)
Dermato/polymyositis	0.25 (43/1,455,995)	0.20 (117/4,444,396)	1.15 (0.78-1.70)	1.11 (0.75-1.65)
Bullous pemphigoid	0.05 (9/1,456,196)	0.04 (25/4,444,868)	1.08 (0.46-2.53)	1.33 (0.55-3.23)
Positive control outcomes				
Myocarditis	0.26 (45/1,456,173)	0.04 (21/4,444,830)	6.84 (3.79-12.35)	6.79 (3.76-12.28)
Pericarditis	0.21 (36/1,456,143)	0.09 (54/4,444,734)	2.15 (1.34-3.46)	2.08 (1.29-3.36)
Guillian-Barre syndrome	0.15 (26/1,456,064)	0.12 (71/4,444,522)	1.14 (0.68-1.91)	1.08 (0.64-1.81)
Negative control outcomes				
Benign skin tumor	28.00 (4,682/1,429,862)	26.31 (14,907/4,398,701)	1.02 (0.98-1.06)	0.99 (0.96-1.03)
Melanoma in situ	0.04 (7/1,456,197)	0.03 (20/4,444,866)	1.12 (0.43-2.93)	1.10 (0.42-2.89)
Tympanic membrane perforation	2.09 (356/1,453,385)	2.36 (1,350/4,439,358)	0.81 (0.71-0.93)	0.79 (0.69-0.90)

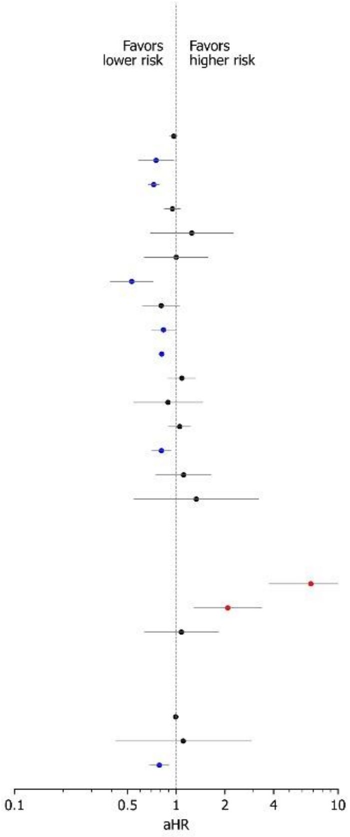

Supplementary Figure 10. Stratified analyses of the risks of incident autoimmune connective tissue disorders in the mRNA-based COVID-19 vaccination cohort compared with the historical control cohort in the subgroup who received only mRNA-based vaccines

The forest plot depicts adjusted hazard ratios (aHRs) with 99% confidence intervals (CIs) in the individuals without history of cross-vaccination with non-mRNA vaccines (BNT162b2 or mRNA-1273) in the mRNA-based COVID-19 vaccination compared with historical controls. The point estimate (centre) represents the aHR, and the horizontal line (error bar) shows the range of the 99% CI. The incidence rate was calculated as the number of events divided by 10,000 person-years, with the population at risk also presented.

Abbreviation: aHR, adjusted hazard ratio; ANCA, antineutrophil cytoplasmic antibody; CI, confidence interval; COVID-19, coronavirus disease 2019; HR, hazard ratio.

	Incidence rate (no. of events/population at risk)			
	**mRNA Vaccination**	**Historical Controls**	HR (99% CI)	**aHR (99% CI)**
Autoimmune outcomes				
Alopecia areata	13.66 (5,422/2,878,470)	13.58 (7,717/4,408,971)	1.07 (1.03-1.11)	1.01 (0.97-1.05)
Cicatricial alopecia	0.57 (228/2,911,401)	0.72 (411/4,442,814)	0.80 (0.67-0.96)	0.79 (0.66-0.94)
Psoriasis	6.51 (2,590/2,885,121)	7.82 (4,450/4,410,397)	0.81 (0.77-0.85)	0.84 (0.80-0.89)
Vitiligo	2.79 (1,118/2,906,214)	2.98 (1,703/4,435,908)	0.90 (0.82-0.98)	0.93 (0.85-1.01)
ANCA-associated vasculitis	0.08 (32/2,913,205)	0.09 (50/4,444,706)	0.83 (0.50-1.38)	0.91 (0.55-1.51)
Sarcoidosis	0.18 (71/2,912,934)	0.17 (97/4,444,347)	1.00 (0.71-1.40)	1.04 (0.74-1.47)
Behcet disease	0.46 (186/2,910,762)	0.66 (380/4,441,279)	0.68 (0.56-0.83)	0.69 (0.57-0.84)
Crohn disease	0.56 (224/2,911,438)	0.61 (350/4,442,829)	0.91 (0.75-1.10)	0.92 (0.76-1.11)
Ulcerative colitis	1.37 (550/2,907,811)	1.47 (840/4,438,134)	0.95 (0.84-1.07)	0.95 (0.84-1.07)
Rheumatoid arthritis	19.93 (7,799/2,843,601)	22.09 (12,399/4,355,972)	0.83 (0.81-0.86)	0.90 (0.87-0.93)
Systemic lupus erythematosus	1.17 (470/2,909,787)	0.91 (520/4,440,824)	1.21 (1.05-1.39)	1.24 (1.08-1.43)
Systemic sclerosis	0.18 (72/2,912,872)	0.16 (92/4,444,267)	1.00 (0.71-1.42)	1.06 (0.75-1.50)
Sjogren syndrome	1.63 (654/2,909,168)	1.45 (831/4,439,578)	1.01 (0.90-1.13)	1.10 (0.98-1.23)
Anklyosing spondylitis	2.24 (898/2,906,187)	2.24 (1,280/4,436,382)	0.96 (0.87-1.05)	1.01 (0.91-1.11)
Dermato/polymyositis	0.22 (89/2,912,919)	0.20 (117/4,444,396)	1.03 (0.76-1.40)	1.11 (0.82-1.52)
Bullous pemphigoid	0.08 (32/2,913,309)	0.04 (25/4,444,868)	1.40 (0.78-2.52)	1.38 (0.76-2.51)
Positive control outcomes				
Myocarditis	0.35 (139/2,913,281)	0.04 (21/4,444,830)	9.12 (5.51-15.10)	9.17 (5.53-15.20)
Pericarditis	0.29 (118/2,913,209)	0.09 (54/4,444,734)	3.06 (2.14-4.38)	3.15 (2.20-4.51)
Guillian-Barre syndrome	0.20 (82/2,913,024)	0.12 (71/4,444,522)	1.54 (1.08-2.21)	1.65 (1.15-2.37)
Negative control outcomes				
Benign skin tumor	27.20 (10,735/2,866,782)	26.31 (14,907/4,398,701)	1.00 (0.97-1.03)	1.03 (1.00-1.06)
Melanoma in situ	0.05 (21/2,913,305)	0.03 (20/4,444,866)	1.34 (0.68-2.64)	1.42 (0.72-2.81)
Tympanic membrane perforation	2.06 (827/2,908,601)	2.36 (1,350/4,439,358)	0.81 (0.74-0.90)	0.87 (0.79-0.96)

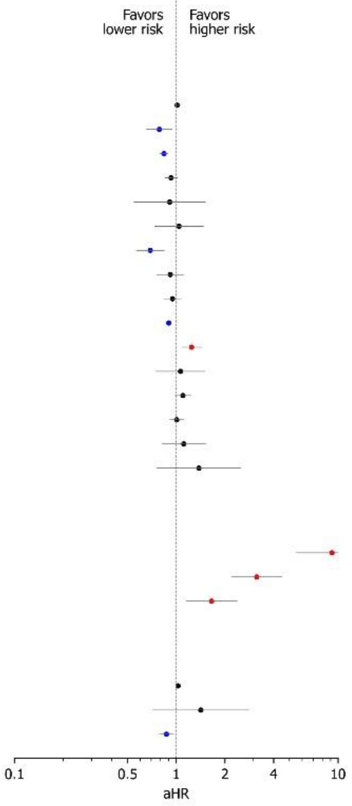

Supplementary Figure 11. Stratified analyses of the risks of incident autoimmune connective tissue disorders in the mRNA-based COVID-19 vaccination cohort compared with the historical control cohort in the subgroup who had history of cross-vaccination with any non-mRNA vaccines

The forest plot depicts adjusted hazard ratios (aHRs) with 99% confidence intervals (CIs) in the individuals with history of cross-vaccination with non-mRNA vaccines (BNT162b2 or mRNA-1273) in the mRNA-based COVID-19 vaccination compared with historical controls. The point estimate (centre) represents the aHR, and the horizontal line (error bar) shows the range of the 99% CI. The incidence rate was calculated as the number of events divided by 10,000 person-years, with the population at risk also presented.

Abbreviation: aHR, adjusted hazard ratio; ANCA, antineutrophil cytoplasmic antibody; CI, confidence interval; COVID-19, coronavirus disease 2019; HR, hazard ratio.

	Incidence rate (no. of events/population at risk)			
	**mRNA Vaccination**	**Historical Controls**	HR (99% CI)	**aHR (99% CI)**
Autoimmune outcomes				
Alopecia areata	11.12 (1,886/1,516,361)	13.58 (7,717/4,408,971)	0.83 (0.78-0.88)	0.97 (0.92-1.03)
Cicatricial alopecia	0.60 (103/1,530,890)	0.72 (411/4,442,814)	0.79 (0.61-1.00)	0.81 (0.63-1.04)
Psoriasis	6.86 (1,163/1,514,634)	7.82 (4,450/4,410,397)	0.79 (0.74-0.85)	0.74 (0.68-0.80)
Vitiligo	3.46 (591/1,526,922)	2.98 (1,703/4,435,908)	1.06 (0.95-1.18)	0.97 (0.87-1.09)
ANCA-associated vasculitis	0.16 (28/1,531,824)	0.09 (50/4,444,706)	1.72 (1.01-2.91)	1.45 (0.85-2.48)
Sarcoidosis	0.23 (40/1,531,647)	0.17 (97/4,444,347)	1.19 (0.78-1.82)	1.09 (0.71-1.68)
Behcet disease	0.52 (89/1,530,259)	0.66 (380/4,441,279)	0.71 (0.55-0.93)	0.70 (0.54-0.92)
Crohn disease	0.61 (104/1,531,091)	0.61 (350/4,442,829)	0.95 (0.74-1.22)	0.96 (0.75-1.24)
Ulcerative colitis	1.50 (257/1,528,729)	1.47 (840/4,438,134)	1.00 (0.86-1.18)	1.02 (0.87-1.20)
Rheumatoid arthritis	23.20 (3,837/1,477,851)	22.09 (12,399/4,355,972)	0.92 (0.89-0.96)	0.80 (0.76-0.83)
Systemic lupus erythematosus	1.03 (176/1,530,021)	0.91 (520/4,440,824)	1.04 (0.85-1.26)	0.99 (0.81-1.21)
Systemic sclerosis	0.18 (31/1,531,626)	0.16 (92/4,444,267)	0.99 (0.62-1.58)	0.88 (0.55-1.41)
Sjogren syndrome	2.01 (344/1,528,707)	1.45 (831/4,439,578)	1.19 (1.03-1.38)	1.02 (0.88-1.18)
Anklyosing spondylitis	2.32 (396/1,527,781)	2.24 (1,280/4,436,382)	0.93 (0.82-1.06)	0.85 (0.75-0.97)
Dermato/polymyositis	0.23 (40/1,531,667)	0.20 (117/4,444,396)	0.99 (0.65-1.49)	0.83 (0.55-1.26)
Bullous pemphigoid	0.10 (17/1,531,892)	0.04 (25/4,444,868)	1.99 (0.99-3.99)	1.98 (0.95-4.13)
Positive control outcomes				
Myocarditis	0.14 (24/1,531,876)	0.04 (21/4,444,830)	3.37 (1.70-6.68)	2.91 (1.45-5.83)
Pericarditis	0.22 (37/1,531,841)	0.09 (54/4,444,734)	2.22 (1.37-3.59)	1.97 (1.21-3.20)
Guillian-Barre syndrome	0.24 (41/1,531,755)	0.12 (71/4,444,522)	1.79 (1.15-2.79)	1.65 (1.05-2.58)
Negative control outcomes				
Benign skin tumor	29.76 (4,998/1,502,045)	26.31 (14,907/4,398,701)	1.08 (1.04-1.12)	1.02 (0.98-1.05)
Melanoma in situ	0.03 (6/1,531,897)	0.03 (20/4,444,866)	0.94 (0.33-2.68)	0.79 (0.27-2.27)
Tympanic membrane perforation	2.27 (389/1,528,526)	2.36 (1,350/4,439,358)	0.88 (0.77-1.00)	0.78 (0.68-0.88)

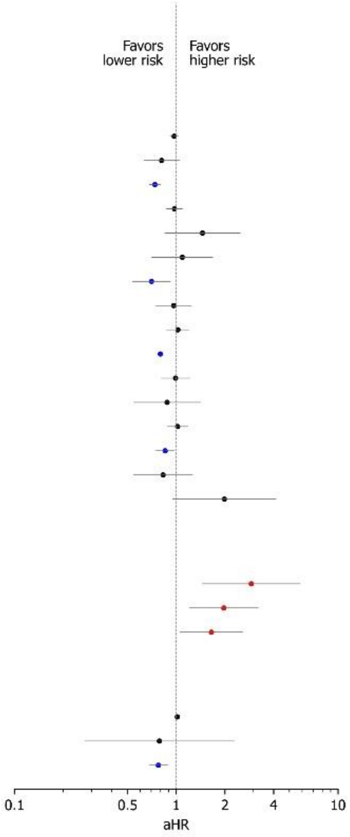

Supplementary Table 1. COVID-19 vaccination profiles of the vaccination cohort

Number of individuals, n (%)	Final vaccine dose		
	**1 dose**	**2 doses**	**3 doses**
Total number	14,979 (100.00)	793,481 (100.00)	3,629,665 (100.00)
Type of mRNA vaccine			
BNT162b2, Pfizer-BioNTech	10,385 (69.33)	469,216 (59.13)	2,438,355 (67.18)
mRNA-1273, Moderna	4,594 (30.67)	321,389 (40.51)	1,126,837 (31.04)
Both		2,876 (0.36)	64,473 (1.78)
Prior non-mRNA vaccination			
No	14,979 (100.00)	628,698 (79.23)	2,269,709 (62.53)
Yes		164,783 (20.77)	1,359,956 (37.47)
ChAdOx1 nCoV-19 (AZD1222), Oxford–AstraZeneca		14,101 (1.78)	1,346,454 (37.10)
Ad26.COV2.S, Janssen-Johnson & Johnson		150,682 (18.99)	13,502 (0.37)

Abbreviation: COVID-19, Coronavirus 2019 disease.

Supplementary Table 2. ***International Statistical Classification of Diseases*, Tenth Revision (ICD-10) codes of the included diseases**

Disease	ICD-10 code
**Autoimmune connective tissue diseases**	
Alopecia areata	L63
Primary cicatricial alopecia	L66
Psoriasis	L40
Vitiligo	L80
Anti-neutrophil cytoplasmic antibody-associated vasculitis	M30.1; M31.3; M31.7; M31.8
Sarcoidosis	D86
Behcet disease	M35.2
Crohn disease	K50
Ulcerative colitis	K51
Rheumatoid arthritis	M05; M06; M08
Systemic lupus erythematosus	M32
Systemic sclerosis	M34
Sjogren syndrome	M35.0
Ankylosing spondylitis	M45
Dermato/polymyositis	M33
Bullous pemphigoid	L120
**Positive control outcomes**	
Myocarditis	I51.4; I40.0; I40.1; I40.8; I40.9
Pericarditis	I30
Guillain-Barre syndrome	G61.0
**Negative control outcomes**	
Benign skin tumor	D23
Melanoma in situ	D03.9
Tympanic membrane perforation	H72
**Chronic diseases served as the covariates**	
Hypertension	I10
Diabetes mellitus	E08; E09; E10; E11; E13
Dyslipidemia	E78
Atopic dermatitis	L20
Allergic rhinitis	J30
Asthma	J45
Hyperthyroidism	E05
Hypothyroidism	E03
Hashimoto thyroiditis	E06.3
Vitamin D deficiency	E55.9
Hepatitis B virus infection	B18.0; B18.1; B191
Hepatitis C virus infection	B18.2; B19.2
Human immunodeficiency virus infection	B20

Abbreviation: ICD-10, International Classification of Diseases, 10th Revision
