# Supplementary material for: Long-term risk of autoimmune diseases after mRNA-based SARS-CoV2 vaccination in a Korean, nationwide, population-based cohort study
Source: Nat Commun. 2024 Jul 23;15:6181. doi: 10.1038/s41467-024-50656-8 (PMC11263712; doi:10.1038/s41467-024-50656-8)
Supplement: Supplementary file 3 — Reporting Summary [file 41467_2024_50656_MOESM3_ESM.pdf]

nature portfolio

Corresponding author(s): Solam Lee

Last updated by author(s): Jun 6, 2024

Reporting Summary

Nature Portfolio wishes to improve the reproducibility of the work that we publish. This form provides structure for consistency and transparency in reporting. For further information on Nature Portfolio policies, see our Editorial Policies and the Editorial Policy Checklist.

Statistics

For all statistical analyses, confirm that the following items are present in the figure legend, table legend, main text, or Methods section.

n/a	Confirmed	
☐	☒	The exact sample size (*n*) for each experimental group/condition, given as a discrete number and unit of measurement
☐	☒	A statement on whether measurements were taken from distinct samples or whether the same sample was measured repeatedly
☐	☒	The statistical test(s) used AND whether they are one- or two-sided<br>*Only common tests should be described solely by name; describe more complex techniques in the Methods section.*
☐	☒	A description of all covariates tested
☐	☒	A description of any assumptions or corrections, such as tests of normality and adjustment for multiple comparisons
☐	☒	A full description of the statistical parameters including central tendency (e.g. means) or other basic estimates (e.g. regression coefficient) AND variation (e.g. standard deviation) or associated estimates of uncertainty (e.g. confidence intervals)
☐	☒	For null hypothesis testing, the test statistic (e.g. *F*, *t*, *r*) with confidence intervals, effect sizes, degrees of freedom and *P* value noted<br>*Give P values as exact values whenever suitable.*
☒	☐	For Bayesian analysis, information on the choice of priors and Markov chain Monte Carlo settings
☒	☐	For hierarchical and complex designs, identification of the appropriate level for tests and full reporting of outcomes
☒	☐	Estimates of effect sizes (e.g. Cohen's *d*, Pearson's *r*), indicating how they were calculated

Our web collection on statistics for biologists contains articles on many of the points above.

Software and code

Policy information about availability of computer code

Data collection	Data collection were performed using SAS (version 9.4; SAS Institute Inc., Cary, NC, USA) for big-data analysis.
Data analysis	Statistical analyses were were performed using R software (version 3.4.1; R Foundation, Vienna, Austria) for generating figures, and SAS (version 9.4; SAS Institute Inc., Cary, NC, USA). An inverse probability of treatment weighting was performed by using propensity score matching alogirhtm provided in the SAS software. Hazard ratios (HRs) with confidence intervals using cox proportional hazard regression models were used for estimation. Stratified subgroup analyses according to sex, age, type of mRNA vaccine, cross-vaccination with non-mRNA vaccine, and COVID-19 diagnosis status. Extended Cox proportional hazard analyses with booster vaccination as time-varying covariate were conducted to account for the variability of vaccination status during the observation.

For manuscripts utilizing custom algorithms or software that are central to the research but not yet described in published literature, software must be made available to editors and reviewers. We strongly encourage code deposition in a community repository (e.g. GitHub). See the Nature Portfolio guidelines for submitting code & software for further information.

Data

Policy information about availability of data

All manuscripts must include a data availability statement. This statement should provide the following information, where applicable:

- Accession codes, unique identifiers, or web links for publicly available datasets
- A description of any restrictions on data availability
- For clinical datasets or third party data, please ensure that the statement adheres to our policy

The datasets analysed during the current study are available in the National Health Insurance Service in South Korea (https://nhiss.nhis.or.kr/bd/ab/bdaba000eng.do). This protects the confidentiality of the data and ensures that Information Governance is robust. Applications to access health data in South Korea are submitted to the National Health Insurance Service in South Korea. Information can be found at https://nhiss.nhis.or.kr/bd/ab/bdaba000eng.do.

Research involving human participants, their data, or biological material

Policy information about studies with human participants or human data. See also policy information about sex, gender (identity/presentation), and sexual orientation and race, ethnicity and racism.

Reporting on sex and gender

We used the population-based nationwide National Health Information Database (Korea Disease Control and Prevention Agency-COIVD-19-National Health Insurance Service cohort [K-COV-N cohort]) in South Korea. Thus, this is determined by the "sex" of a participant reported to the government system.

Reporting on race, ethnicity, or other socially relevant groupings

For the study, the claims based data of South Korea were used to comprehensively investigate the association of the study outcome.

We utilized socially relevant variables officially reported in K-COV-N cohort database, which were categorized based on prior research that considered biases in healthcare access and social environments.

- Main cohort: insurance type (standard and medicaid); income level quartiles (lowest [0–24], lower [25–49], higher [50-74], and highest [75–100]); region of residence (urban and rural)

Following variables were used to adjust confounding factors in the study:
- Main cohort: age (<40, 40–59, and ≥60 years); sex; insurance type (standard and medicaid), income level (highest, higher, lower, and lowest income divided into quartiles based on health insurance premiums), area of residence (urban or rural), underlying diseases (hypertension, diabetes mellitus, dyslipidemia, atopic dermatitis, allergic rhinitis, asthma, hypothyroidism, hyperthyroidism, Hashimoto thyroiditis, vitamin D deficiency, hepatitis B, hepatitis C, and HIV infection), smoking status (current smoker), and alcohol consumption (drinking as routine alcohol consumption, regardless of the amount or frequency).

Population characteristics

The dataset was linked and consisted of data on general health examination results, death records, health insurance data including insurance eligibility data, personal sociodemographic data, inpatient and outpatient healthcare records, and medication records.

- Main cohort: age (<40, 40–59, and ≥60 years); sex; insurance type (standard and medicaid), income level (highest, higher, lower, and lowest income divided into quartiles based on health insurance premiums), area of residence (urban or rural), underlying diseases (hypertension, diabetes mellitus, dyslipidemia, atopic dermatitis, allergic rhinitis, asthma, hypothyroidism, hyperthyroidism, Hashimoto thyroiditis, vitamin D deficiency, hepatitis B, hepatitis C, and HIV infection), smoking status (current smoker), and alcohol consumption (drinking as routine alcohol consumption, regardless of the amount or frequency).

Recruitment

We used the population-based nationwide National Health Information Database (K-COV-N cohort) in South Korea, and medical records that can identify past medical history before the index date and incident events during the observation period have been available since January 1, 2016. The study included participants who underwent a general health examination in 2018 (n=15,076,899). We randomly selected 9,945,450 participants, according to the data regulation and policy for the K-COV-N cohort database. After excluding participants (n = 80,556) with incomplete general health examination reports, the primary cohort comprised 9,258,803 individuals vaccinated with at least one dose of an mRNA-based COVID-19 vaccine until December 31, 2022. We subsequently extracted half of the primary cohort to establish the vaccination cohort, whose index was the date of administration of the first dose of the mRNA-based COVID-19 vaccine. The other half of the primary cohort was used to form historical control cohorts, and the observational period for the control group was shifted back by 2 years from the date of the first dose of mRNA vaccination. The two cohort groups were followed up from the respective study index date to disease diagnosis, emigration, death, or the end of the study period. The vaccination group was observed until December 31, 2022, and the historical cohort group until December 31, 2020. The final sample sizes were 4,445,333 participants in the vaccination cohort and 4,444,932 participants in the historical control cohort.

Ethics oversight

This study received approvals from the the Korea Disease Control and Prevention Agency (KDCA) and National Health Insurance (NHIS; KDCA-NHIS-2023-1-500). Under the terms of the approval, patient consent was not required for the use of routine health records for our study.

Note that full information on the approval of the study protocol must also be provided in the manuscript.

Field-specific reporting

Please select the one below that is the best fit for your research. If you are not sure, read the appropriate sections before making your selection.

- [x] Life sciences
- [ ] Behavioural & social sciences
- [ ] Ecological, evolutionary & environmental sciences

For a reference copy of the document with all sections, see nature.com/documents/nr-reporting-summary-flat.pdf

Life sciences study design

All studies must disclose on these points even when the disclosure is negative.

Sample size

This study utilized nationwide population-based cohorts, including a South Korean claims-based nationwide cohort (K-CoV-N; N=15,076,899) who underwent a general health examination in 2018.

Data exclusions

Among 15,076,899 participants in South Korea, we randomly selected 9,945,450 participants, approximately 20% of the total population of South Korea according to the data regulation and policy for the K-COV-N cohort database. We excluded participants (n = 80,556) with incomplete general health examination reports. The sample size of primary cohort was 9,258,803 individuals Individuals vaccinated with at least one dose of mRNA-based COVID 19 vaccine (either BNT162b2 or mRNA-1273) until Dec 31, 2022. We subsequently extracted half of the primary cohort to establish the vaccination cohort, and the other half was used to form historical control cohorts. The final sample sizes were 4,445,333 participants in the vaccination cohort and 4,444,932 participants in the historical control cohort, respectively.

Replication

We used methodologies with propensity score and inverse probability treatment weighting (IPTW) to determine robustness and generalization of our main results. Models were adjusted for following variables:

- Model: Adjusted for age (<40, 40–59, and ≥60 years); sex; insurance type (standard and medicaid), income level (highest, higher, lower, and lowest income divided into quartiles based on health insurance premiums), area of residence (urban or rural), underlying diseases (hypertension, diabetes mellitus, dyslipidemia, atopic dermatitis, allergic rhinitis, asthma, hypothyroidism, hyperthyroidism, Hashimoto thyroiditis, vitamin D deficiency, hepatitis B, hepatitis C, and HIV infection), smoking status (current smoker), and alcohol consumption (drinking as routine alcohol consumption, regardless of the amount or frequency).

A Cox proportional hazards analysis after adjusting for all predefined covariates used to calculate the IPTW, with estimates of HRs and adjusted 99% CIs was used to explore incident autoimmune connective tissue diseases associated with mRNA-based COVID-19 vaccinations. To consider potential impacts of several confounding factors, stratified subgroup analyses were conducted according to sex, age (<40 vs. ≥40), type of mRNA vaccine (BNT162b2 vs. mRNA-1273), cross-vaccination with non-mRNA vaccine (ChAdOx1 nCoV-19 (AZD1222) or Ad26.COV2.S), and COVID-19 diagnosis status. Additionally, we conducted extended Cox proportional hazard analyses with booster vaccination as time-varying covariate to account for its variability during the observation.

Randomization

Not applicable; individuals were recruited in an observational study with convenience samples being collected.

Blinding

Blinding was not relevant, since this is an observational study, where participants were invited based on test status (case or control).

Reporting for specific materials, systems and methods

We require information from authors about some types of materials, experimental systems and methods used in many studies. Here, indicate whether each material, system or method listed is relevant to your study. If you are not sure if a list item applies to your research, read the appropriate section before selecting a response.

Materials & experimental systems

n/a	Involved in the study
x	Antibodies
x	Eukaryotic cell lines
x	Palaeontology and archaeology
x	Animals and other organisms
x	Clinical data
x	Dual use research of concern
x	Plants

Methods

n/a	Involved in the study
x	ChIP-seq
x	Flow cytometry
x	MRI-based neuroimaging

Plants

Seed stocks	Not applicable.
Novel plant genotypes	Not applicable.
Authentication	Not applicable.
